# Exploring the link between AI usage intention and digital competence among college PE teachers: A moderated mediation model based on SCT and UTAUT

**DOI:** 10.1371/journal.pone.0334699

**Published:** 2025-11-21

**Authors:** Rong Yi, Dinghua Liu, Xu Sun, Bo Zhou

**Affiliations:** 1 College of Physical Education, Hunan Normal University, Changsha, China; 2 College of Physical Education, Hunan University, Changsha, China; Zhejiang Normal University, CHINA

## Abstract

As artificial intelligence (AI) technologies rapidly integrate into higher education, they impose increasing demands on the teaching approaches and digital competence of physical education teachers. However, the relationship between physical education teachers’ behavioral intention to use AI and their digital competence remains underexplored. This study focuses on college physical education teachers and examines the relationship between their intention to use AI and their digital competence. Grounded in Social Cognitive Theory (SCT) and the Unified Theory of Acceptance and Use of Technology (UTAUT), the study proposes a structural equation model incorporating behavioral intention, self-efficacy, social influence, and digital competence, with gender as a moderating variable. A questionnaire survey was conducted among 479 physical education teachers from ten universities in mainland China, and the model was tested using AMOS and SPSS. The results indicate that teachers’ behavioral intention to use AI is positively associated with their self-efficacy, perceived social influence, and digital competence, with both self-efficacy and social influence serving as significant mediators. Furthermore, self-efficacy is positively related to social influence, while gender does not exert a significant moderating effect on any of the proposed paths. This study contributes to the integrated application of SCT and UTAUT in the context of physical education in higher education and offers theoretical and practical implications for enhancing digital competence and promoting intelligent transformation among college physical education teachers.

## Introduction

In recent years, artificial intelligence (AI) technologies have rapidly permeated the field of higher education, driving profound transformations in teaching and learning [1]. A U.S.-based survey reported that approximately 40% of teachers use ChatGPT weekly and 10% use it daily, primarily for lesson planning (30%), classroom design (30%), and providing supplementary background knowledge (27%) [2]. In parallel, China’s national policy document Educational Modernization Plan (2024–2035) explicitly calls for the digital upgrading of academic disciplines and the transformation of research paradigms, aiming to optimize the structure of higher education. However, successful AI integration in university teaching depends largely on teachers’ attitudes and their willingness to adopt these technologies [3,4]. Many educators remain hesitant or unprepared to incorporate AI into their teaching practices, and institutional integration remains relatively slow [5,6]. Against this backdrop, the acceptance of AI technologies by educators and its influencing factors has emerged as a focal point in educational technology research.

Several studies have adopted structural equation modeling to empirically examine the factors shaping teachers’ and students’ intentions to use AI [7]. Wu et al. [8] found that university students’ acceptance of AI-assisted learning environments is significantly influenced by performance expectancy, effort expectancy, and social influence, while multidimensional perceived risk negatively affects their attitudes. In contrast, Chatterjee and Bhattacharjee [9] focused on university teachers and found that effort expectancy and facilitating conditions positively predicted intention, while performance expectancy had no significant effect, indicating that educators place greater emphasis on accessibility and support infrastructure. In specific domains, the application of AI in education is expanding, including accounting [10], medical education [11], and engineering education [12]. In the digital era, AI technologies are increasingly seen as enablers of educational innovation, and teachers’ digital competence (TDC) has become a fundamental prerequisite for achieving high-quality education [13]. TDC is a multifaceted construct encompassing technological skills, pedagogical knowledge, instructional design, ethical awareness, and professional growth, and represents a teacher’s capacity to teach effectively and responsibly in digital contexts [14]. Whether teachers possess adequate digital competence to flexibly and effectively integrate AI into their teaching and support students’ adaptation to intelligent learning environments is now recognized as a critical factor in the successful adoption of emerging technologies [15].

Physical education teacher relies heavily on embodied practice, intensive interaction, and targeted feedback, which presents unique challenges for AI integration [16]. Existing research has explored the role of AI in physical education instruction [17], physical activity interventions [18], exercise addiction [19], and sports industry development [20]. However, empirical studies specifically examining university PE teachers’ behavioral intention (BI) to adopt AI technologies remain scarce. At the same time, traditional social cognitive theory (SCT) and the Unified Theory of Acceptance and Use of Technology (UTAUT) emphasize the influence of self-efficacy on behavioral intentions in the application of technology adoption pathways [21,22]. Previous study has shown that college students’ computer self-efficacy significantly predicts their willingness to use AI translation technology [23]. But in the current context of educational intelligence, university physical education teachers have demonstrated greater technological sensitivity and proactive adaptation. Their acceptance of AI is not only reflected in their willingness to use it, but also accompanied by continuous improvement in their digital competence. This research gap could theoretically provide new insights for traditional theories and practical experience for promoting the professional development of college physical education teachers. Its significance lies in its positive implications for improving the AI teaching abilities of college physical education teachers and optimizing the institutional environment.

To address this research gap, this study focuses on the relationship between college physical education teachers’ willingness to use AI and their own digital competence, attempting to break through the single perspective of traditional theoretical models and re-examine the driving effect of behavioral intention as a positive cognitive tendency on the construction of teachers’ digital competence, thereby expanding the explanatory boundaries of the theory. The purpose of this study is to systematically clarify the current status of college physical education teachers’ willingness to use AI in teaching contexts. Based on an integrated perspective of SCT and UTAUT, a predictive path model centered on behavioral intention is constructed to empirically test its mechanisms of influence on self-efficacy (SE), social influence (SI), and TDC, thereby revealing the moderating effect of gender factors in this mechanism. This study aims to provide theoretical support and practical evidence for promoting teachers’ AI capability development. Furthermore, the study adopted a questionnaire survey format, with physical education teachers from mainland Chinese universities as the sample population. Structural equation modeling was used to validate the structural relationships between relevant variables, providing empirical support and strategic recommendations for the precise enhancement of teachers’ professional capabilities in the context of the intelligent development of physical education.

## Literature review

### Theoretical foundation

The relationship between college physical education teachers’ intention to use AI and their digital competence is shaped by a complex interplay of multiple factors. To explain and predict this relationship, it is essential to draw upon robust theoretical foundations. In this study, we adopt SCT and UTAUT as the primary theoretical frameworks. These two theories, respectively emphasizing individual cognition, environmental interaction, and technology acceptance factors, offer valuable perspectives for understanding how behavioral intention toward AI use relates to digital competence among college physical education teachers.

### Social cognitive theory

SCT, first proposed by Bandura in 1986, serves as a foundational framework for understanding human motivation, learning, and behavioral change [21]. SCT has been widely applied across diverse domains, including education, health behavior, organizational dynamics, and technology adoption [24,25]. The theory proposes a triadic reciprocal causation model, where personal factors, behaviors, and environmental influences continuously interact. At the core of SCT is the construct of self-efficacy, defined as an individual’s belief in their capability to perform a specific behavior, which is regarded as a key determinant of behavioral intention and persistence [24]. By emphasizing human agency and the cognitive processes embedded in social contexts, SCT has evolved into a robust psychological mechanism for explaining how individuals adopt and sustain the use of new technologies.

In recent years, scholars have begun applying SCT to physical education contexts. For instance, Li et al. [26] identified innovation as a critical factor in the development of physical education and found that teachers’ self-efficacy significantly influences their psychological workload, decision-making processes, and intention to innovate. Similarly, Lin and Zhu [27] demonstrated that self-efficacy is a key factor influencing students’ interest in learning physical education. Students with higher self-efficacy tend to show greater enthusiasm for physical education. Despite its widespread application in educational technology research, empirical studies using SCT in the field of physical education remain limited. Physical education teaching is deeply contextualized and embodied, requiring intense physical interaction and real-time feedback. As such, physical education teachers’ adoption of digital technologies like AI is shaped not only by cognitive judgments but also by environmental support and experiential constraints [28].

Given this complexity, the formation of physical education teachers’ behavioral intentions toward AI adoption remains insufficiently understood. Emerging research suggests that such intentions may be influenced by a combination of digital competence, familiarity with technological platforms, and institutional support systems [29]. These findings highlight the limitations of relying solely on cognitive predictors to explain teachers’ AI adoption behavior. Although classical theoretical frameworks have predominantly emphasized the predictive influence of SE on BI, emerging evidence suggests the possibility of a dynamic or even reversed causal relationship between these two constructs [30]. In certain specific behavioral contexts, a clearly formed intention to act may, in turn, reinforce individuals’ perceptions of their own competence and confidence. This view is supported by the following mechanisms: (a) Psychological Empowerment Theory: Once individuals develop a strong behavioral intention, it may activate their intrinsic motivation and sense of agency, thereby enhancing perceived self-efficacy [31]. (b) Self-enhancement and Feedback Mechanisms: When a person is already motivated to perform a behavior, they are more likely to engage in preparatory actions, seek relevant resources, and formulate action plans. These proactive behaviors can contribute to a heightened sense of capability and control [32,33]. Therefore, while we do not dismiss the theoretical soundness of the traditional SE to BI pathway, we argue that, within the particular empirical context of the present study, the alternative path from BI to SE in college physical education is both conceptually grounded and worthy of scholarly consideration.

### UTAUT theory

UTAUT, proposed by Venkatesh et al. [22], aims to integrate key theoretical perspectives in the domain of technology adoption into a unified and more predictive model. Drawing on core constructs from established frameworks such as the Technology Acceptance Model Theory of Planned Behavior, and Innovation Diffusion Theory, UTAUT examines how users’ perceptions and attitudes influence their behavioral responses toward emerging technologies. The model identifies four primary determinants of technology acceptance: performance expectancy, effort expectancy, social influence, and facilitating conditions. Among these, the first three primarily predict BI, while facilitating conditions that directly influence actual usage. Additionally, the model incorporates four moderators: gender, age, experience, and voluntariness of use. These factors to account for individual differences in these relationships [22].

Due to its clarity and generalizability, UTAUT has been widely validated across domains such as higher education [34], healthcare [35], and architecture [36]. Within the model, BI serves as the central outcome variable to gauge individuals’ willingness to adopt new technologies, while SI has been consistently shown to exert a significant positive effect on BI [37]. In the context of mobile payment, for example, SI was found to significantly predict users’ adoption intentions, which in turn influenced actual usage behavior [38]. Notably, SI is closely linked with SE, as encouragement and social support from others can enhance users’ confidence in engaging with technology [39], thus suggesting a potential pathway connecting these constructs.

In UTAUT, SI is traditionally conceptualized as an antecedent of BI. However, considering the specific context of university physical education teachers and the multifaceted nature of their attitudes toward adopting AI technologies, this study proposes a reverse pathway from BI to SI and provides justification from two complementary perspectives. First, from the standpoint of experiential learning and feedback mechanisms, once teachers develop a clear intention to use AI (i.e., high BI), they are more likely to actively seek peer support, technical guidance, and social recognition. Second, based on the theory of reciprocal influence, BI is not only shaped by external social factors but can also modulate an individual’s sensitivity to and interpretation of those social cues. For instance, when college PE teachers openly express a strong intention, their social counterparts may respond with supportive behaviors, which in turn contributes to a more socially reinforcing environment [40]. Therefore, although the original UTAUT model adopts a unidirectional SI to BI structure, we argue that within the initial stage of AI adoption among university physical education teachers, BI can serve as a meaningful predictor of SI. This alternative pathway holds both theoretical plausibility and contextual relevance.

In summary, considering that college physical education teachers exhibit a high degree of instructional autonomy while also being influenced by organizational policies, prevailing social norms, and peer expectations, this study integrates SCT and UTAUT to construct a more adaptable and explanatory theoretical framework. SCT emphasizes individual self-regulation and cognitive motivation, with particular attention to the role of SE in shaping behavioral intention, making it well-suited to capture the internal drivers behind teachers’ AI adoption decisions. In contrast, UTAUT focuses on the influence of external social factors, such as SI and performance expectancy, thereby complementing SCT by accounting for contextual determinants of behavioral intention in educational settings. The joint application of these frameworks not only enhances the overall fit and explanatory power of the proposed structural equation model but also offers a theoretically grounded and empirically testable approach for analyzing AI adoption behaviors. This integrative perspective contributes to both theoretical enrichment and practical insights in the context of AI integration within higher physical education.

### Development and assessment of teachers’ digital competence

TDC typically refers to the integrated knowledge, skills, and attitudes necessary for the effective use of digital technologies in educational contexts. Internationally, the concept is most commonly grounded in the European Commission’s “Digital Competence Framework for Educators” (DigCompEdu), which emphasizes the pedagogical integration of digital technologies rather than merely technical proficiency [41]. Unlike traditional models focused on operational skills, DigCompEdu highlights how technology can enhance and transform teaching and learning. Its structured and adaptable framework has demonstrated applicability across diverse educational levels, from early childhood to higher education, offering a robust theoretical and practical basis for assessing and developing digital competence among university physical education teachers.

A growing body of research has explored TDC across disciplines and countries, revealing a rich array of findings. Most research relies on self-reported measures and views digital competence as a multidimensional construct including knowledge, technical skills, and attitudinal readiness [42]. Empirical evidence consistently shows significant differences in competence levels across subject areas and educational stages [43]. For example, a large-scale survey of Portuguese STEM teachers found that biology teachers scored significantly higher in digital competence than their peers in mathematics and other subjects [44]. Regionally, countries differ in their emphasis and progress regarding the development of teachers’ digital skills. In China, digital competence among university teachers is often assessed using the TPACK model or local ICT competency standards. Studies have identified various influencing factors, including personal attributes (e.g., age, digital confidence) and contextual conditions (e.g., policy support, infrastructure) [45].

Despite this progress, research on digital competence among physical education teachers remains limited. Studies from Spain, for example, indicate that most primary school physical education teachers demonstrate relatively low levels of digital teaching capability [46]. Moreover, generational disparities are evident: younger physical education teachers generally exhibit stronger digital proficiency than older ones [47]. External constraints such as inadequate digital infrastructure and limited professional training opportunities are also frequently cited as major barriers [48]. With the increasing incorporation of AI technologies in PE instruction, the question of whether university physical education teachers possess the necessary digital competence to effectively use such technologies is becoming increasingly critical. However, empirical studies that examine the relationship between AI use and digital competence in this context remain scarce, highlighting a meaningful and timely research gap.

### Conceptual model and hypotheses

**Fig 1:**
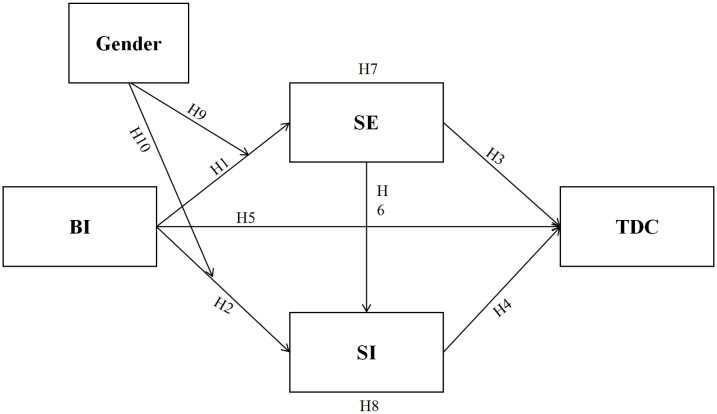
Conceptual model.

To further explore the relationship between university physical education teachers’ willingness to use AI and their digital competence, this study integrates SCT and UTAUT to develop a comprehensive theoretical model (Fig 1). This model incorporates direct effects, dual mediation effects, and gender as a moderating variable. Specifically, teachers’ AI usage intention is conceptualized as the antecedent variable, while SE and perceived SI function as mediators influencing digital competence. Gender is introduced as a moderator to examine whether it affects the strength or direction of these pathways. By establishing this integrated model, the study aims to clarify the underlying mechanisms linking AI usage intention, SE, SI, and TDC, thereby shedding light on how physical education teachers’ willingness to adopt AI may impact their digital competence in the context of higher education.

### Behavioral intention, self-efficacy, social influence, teachers’ digital competence

(1) Behavioral intention and self-efficacy

Prior research has consistently demonstrated that BI serves as a proximal predictor of actual engagement behaviors in technology adoption contexts [22]. When teachers hold strong intentions to integrate AI into their instruction, they are more likely to seek learning opportunities, engage in trial-and-error practices, and interact with digital tools, all of which are known contributors to enhancing self-efficacy beliefs [47]. In particular, physical education teachers with high AI usage intention may accumulate positive experiences in lesson planning, data interpretation, or feedback generation through AI systems, reinforcing their confidence in using such technologies [48]. This aligns with the tenet of SCT, which posits that intentional actions and sustained effort are essential mechanisms for developing perceived competence [25]. Accordingly, we hypothesize that:

H1: College physical education teachers’ behavioral intention to use AI positively influences their self-efficacy.

(2) Behavioral intention and social influence

Teachers who exhibit strong behavioral intention to adopt AI technologies are more likely to engage in visible, consistent, and proactive usage behaviors, which, in turn, shapes how others within their professional environment perceive and respond to such adoption efforts. In organizational settings such as universities, individuals with high intention to use educational technology tend to receive increased social recognition, encouragement, and normative support from peers, administrators, and students [49]. As BI translates into observable actions, such as AI tool implementation in lesson planning or assessment, teachers’ practices become a model for others, thereby reinforcing perceived SI through peer affirmation and institutional endorsement [50]. Particularly in the context of university physical education teaching, where AI is an emerging but not yet normalized instructional tool, early adopters with strong intention may experience heightened social expectations and validation, further solidifying their perception of SI. Based on these considerations, we propose the following hypothesis:

H2: College physical education teachers’ behavioral intention to use AI positively influences their perceived social influence.

(3) Self-efficacy and teachers’ digital competence

Teachers with higher levels of AI-related self-efficacy are more likely to approach technology integration with confidence, persistence, and proactive learning behaviors, which are foundational to the development of digital competence. In particular, SE positively influences teachers’ motivation to experiment with emerging tools, seek feedback, and resolve technical challenges independently [51]. In university physical education contexts, where the integration of AI remains in its early stages, such confidence becomes essential for teachers to explore AI-based instructional design, performance analytics, and student-adaptive feedback systems. As they gain experience through successful implementation, their digital competence. It is defined as the ability to select, apply, and evaluate digital tools for pedagogical purposes [52]. Moreover, studies show that teachers’ self-belief in managing AI technologies can predict their willingness to adopt innovations in instructional contexts, which in turn contributes to broader competence development [53]. Based on this reasoning, we propose the following hypothesis:

H3: College physical education teachers’ self-efficacy in using AI positively influences teacher digital competence.

(4) Social influence and teacher digital competence

SI has been found to play a crucial role in shaping teachers’ engagement with digital innovations [54]. When college physical education teachers perceive strong support or expectations from colleagues, administrators, and institutional policies concerning AI adoption, they are more likely to conform to these normative pressures. This perceived encouragement not only reinforces their willingness to experiment with AI technologies but also fosters continuous learning and skill acquisition. Empirical studies have confirmed that higher levels of perceived social influence are associated with increased motivation to integrate digital tools, which in turn contributes to the development of digital competence [55]. In the context of university physical education, where AI-supported instruction is still emerging, teachers who sense a supportive culture around AI use, such as recognition from peers or endorsements from school leadership, tend to adopt AI more confidently and innovatively, ultimately enhancing their ability to embed such technologies effectively into their teaching practices [56]. Thus, we hypothesize:

H4: College physical education teachers perceived social influence in using AI positively influences teacher digital competence.

(5) Behavioral intention and teacher digital competence

BI is widely recognized as a central psychological predictor of technology adoption in education. Research in STEM education has shown that educational innovations are not only associated with students’ competency development but also with their career aspirations, indicating that the interaction between competence and intention is a pervasive phenomenon in education [57]. When college physical education teachers possess a strong intention to use AI, they are more likely to engage in continuous exploration, experimentation, and integration of AI tools into their teaching routines. This proactive engagement fosters opportunities to practice, reflect, and refine their digital pedagogical strategies, ultimately enhancing their digital competence. Empirical studies have shown that teachers with stronger behavioral intention toward technology use exhibit higher levels of confidence and adaptability in applying digital tools for instruction [58]. Particularly in higher education contexts, intentional engagement with educational technology has been found to be a significant driver of professional digital competence development [59]. In the evolving landscape of AI-supported physical education, where technological demands intersect with instructional innovation, behavioral intention acts not only as a motivational catalyst but also as a developmental pathway for improving digital proficiency. Based on this reasoning, we propose the following hypothesis:

H5: College physical education teachers’ behavioral intention to use AI positively influences teacher digital competence.

(6) Self-efficacy and social influence

Teachers’ self-efficacy in using artificial intelligence shapes how others perceive them within their professional community. Educators with high SE tend to act as initiators of technological innovation, which positions them as opinion leaders or role models in their institutional environment. This elevated visibility often translates into increased recognition and approval from colleagues, administrators, and even students, key sources of SI in educational settings. Empirical research has shown that teachers with higher digital SE are more likely to engage in professional discourse, share best practices, and influence peer behavior regarding technology use [60]. In contexts where AI is being introduced into physical education, those who are confident in their ability to use AI are more likely to attract positive social expectations and encouragement, which strengthens their perceived SI. Moreover, SE plays a critical role in forming social normative beliefs, particularly in collaborative environments where peer endorsement and administrative support are central to innovation adoption [61]. Hence, it is reasonable to hypothesize the following:

H6: College physical education teachers’ self-efficacy in using AI positively influences their perceived social influence.

### Mediating effects of self-efficacy and social influence

In the context of higher education physical education instructors, the relationship between their BI to use AI and their technological digital competence is influenced by multiple factors. A focus solely on direct effects may fail to fully explain the underlying mechanisms, necessitating an exploration of potential mediating variables.

First, SE, as a critical psychological mechanism, may serve as a bridge between BI and TDC. Research in educational technology shows that self-efficacy significantly influences teachers’ technology adoption behaviors. For instance, An et al. [62] found that technological self-efficacy mediated the relationship between technology acceptance and student autonomous learning, suggesting that individuals’ confidence in technology can enhance their motivation and proactive learning behaviors. Similarly, in physical education settings, teachers’ self-efficacy plays a vital role in technology integration. Studies show a significant positive correlation between physical education teachers’ technological self-efficacy and their technology integration ability. Factors such as technical training and social persuasion positively influence self-efficacy [63]. Therefore, as an intrinsic psychological mechanism, self-efficacy may mediate the relationship between physical education instructors’ AI usage intention and digital competence. When teachers develop a positive intention to use AI, their self-efficacy can drive active learning and practice of AI technologies, thereby enhancing their digital competence.

H7: College physical education teachers’ self-efficacy mediates the relationship between their behavioral intention to use AI and teacher digital competence.

Second, SI is regarded as a key external mechanism affecting teachers’ technology adoption behaviors. Research suggests that social influence not only directly strengthens BI but also indirectly facilitates technology adoption by boosting confidence and readiness. For example, in higher education, social factors such as peer recommendations and institutional support have been found to significantly influence teachers’ adoption of educational technology [64]. In physical education teaching, studies indicate that during the COVID-19 pandemic, SI significantly affected physical education teachers’ behavioral intention toward remote teaching [65]. Additionally, Saiz-González et al. [66] highlighted that physical education teachers primarily rely on peer advice and informal learning channels when integrating technology, underscoring the importance of SI in the technology adoption process. Thus, when physical education instructors develop a positive intention to use AI, SI from colleagues, leaders, and institutions can enhance their confidence and readiness, thereby improving their digital competence. This underscores the mediating role of social influence between physical education teachers’ AI usage intention and digital competence.

H8: College physical education teachers perceived social influence mediates the relationship between their behavioral intention to use AI and teacher digital competence.

### The moderating effects of gender

In the context of AI-assisted instruction, gender (as a key demographic variable) may significantly moderate individuals’ psychological responses and social influences. Both the UTAUT model and SCT provide theoretical frameworks for understanding gender differences [22,67].

Research on educational technology adoption has extensively explored gender disparities. For instance, Xia et al. [68] found that in AI-assisted learning environments, female students reported significantly higher satisfaction in psychological needs (e.g., autonomy and competence) than males, with gender exhibiting a notable moderating effect on self-regulated learning (SRL) processes. This suggests that females may perceive psychological variables more sensitively in AI system usage, where extrinsic motivations more readily translate into learning drivers.

Among educators, Scherer and Siddiq [69] observed that while male teachers scored higher in self-efficacy for basic and advanced computer operations, no significant gender differences emerged in teaching-oriented technology use self-efficacy.

Overall, existing studies generally support gender’s moderating role in technology adoption. However, current research primarily focuses on K-12 or general academic settings, with limited systematic empirical investigation into higher education PE instructors’ behavioral intentions toward AI technology. Thus, this study hypothesizes that:

H9: Gender moderates the relationship between college physical education teachers’ behavioral intention to use AI and their self-efficacy.

H10: Gender moderates the relationship between college physical education teachers’ behavioral intention to use AI and their perceived social influence.

## Materials and methods

### Research instruments

The measurement scales used in this study were adapted from existing validated instruments and modified to fit the specific context of AI adoption behavioral intentions among university physical education instructors. All variables were measured using a 7-point Likert scale ranging from 1 (strongly disagree) to 7 (strongly agree).

(1) Behavioral Intention (BI)

BI reflects the degree to which university physical education instructors are willing to adopt AI-assisted teaching technologies. This construct was measured using three items adapted from UTAUT by Venkatesh et al. [22], tailored to the context of AI-assisted physical education instruction. In the current study, the scale demonstrated good reliability and validity (Cronbach’s α = 0.831; McDonald’s ω = 0.899; CR = 0.832; AVE = 0.623).

(2) Self-Efficacy (SE)

SE represents teachers’ confidence in their ability to use AI tools effectively in teaching. Three items were adopted from Chao [70], grounded in Bandura’s SCT [21]. In the study, the items were modified to reflect AI usage in the context of physical education teaching (Cronbach’s α = 0.820; ω = 0.893; CR = 0.821; AVE = 0.604).

(3) Social Influence (SI)

SI was measured using four items adapted from the UTAUT model, focusing on perceived encouragement from colleagues, administrators, and the broader educational environment toward AI integration [71]. The measurement exhibited strong psychometric properties in this study (α = 0.864; ω = 0.908; CR = 0.864; AVE = 0.615).

(4) Teacher’s Digital Competence (TDC)

Teacher’s Digital Competence refers to the ability of physical education instructors to integrate digital tools and resources effectively into instructional practice. It was assessed using ten items adapted from the DigComp2.1 framework [72]. The instrument demonstrated excellent reliability and convergent validity (Cronbach’s α = 0.931; ω = 0.942; CR = 0.931; AVE = 0.575).

### Data collection

To ensure the scientific validity of the sample size for hypothesis testing, this study employed G*Power 3.1 software [73] for sample size estimation. With three predictor variables, a significance level of α = 0.05, the statistical power of 0.95, and an anticipated medium effect size (f^2^ = 0.15), the minimum sample size calculated by G*Power was 119 participants. Additionally, referencing classical recommendations in structural equation modeling (SEM) methodology suggesting a minimum sample size of 200 [74], and accounting for potential invalid responses and missing data during questionnaire administration, the study ultimately set the target sample size at no fewer than 200 to enhance the robustness of parameter estimation and the statistical reliability of the findings.

Participants were recruited between June 1, 2025 and June 7, 2025 through the Wenjuanxing online survey platform (www.wjx.cn). Prior to accessing the questionnaire, all participants were presented with a bilingual electronic informed consent statement outlining the purpose of the study, the anonymous and confidential nature of data collection, and their rights to voluntary participation and withdrawal. Participants provided informed consent electronically by proceeding to complete the questionnaire. The study did not include minors, therefore, parental or guardian consent was not required. A combination of stratified and convenience sampling was employed, with universities categorized into comprehensive and sports-specialized institutions. Ten universities (five comprehensive and five sports-specialized) in mainland China were selected, and physical education teachers from these institutions were invited to participate via faculty communication platforms (e.g., WeChat and QQ groups). The study was approved by the Ethics Committee of Hunan Normal University (Approval No.: [2025507]), with all procedures involving human participants adhering to the principles outlined in the Declaration of Helsinki.

To ensure linguistic accuracy and conceptual equivalence in scale translation, the research team employed back-translation and expert review procedures, with three bilingual researchers (including one professor of physical education) independently translating the items followed by multiple rounds of discussion and revision to finalize the Chinese version. Prior to formal data collection, the research team conducted a pilot test with 25 university physical education instructors and refined the wording of certain items based on feedback to improve clarity and response experience, with pilot test data excluded from the final analysis. A total of 502 questionnaires were collected (Table 1), with 479 valid responses retained after excluding invalid entries, yielding an effective response rate of 95.42%. Among the valid responses, 220 (45.9%) were from male participants and 259 (54.1%) from female participants, indicating a relatively balanced gender distribution. The highest behavioral intention was reported for text-generation AI technologies (e.g., Deepseek) at 73.1%, followed by image generation (64.7%), audio generation (59.5%), and video generation (53.0%). These results demonstrate strong behavioral intentions among university physical education teachers toward AI adoption, particularly for text-generation AI tools.

**Table 1:** The demographic information of the samples.

Variables	Description	N	%
Gender	Male	220	45.9
	Female	259	54.1
Types of AI applications	Text generation (e.g., Deepseek)	350	73.1
	Image generation (e.g., Midjourney)	310	64.7
	Video generation (e.g., Sora)	254	53.0
	Audio generation (e.g., Jukebox)	285	59.5

### Data analysis

First, exploratory factor analysis (EFA) was conducted using SPSS 26.0 to preliminarily examine the underlying factor structure of each scale. The suitability of data for factor analysis was assessed through the Kaiser-Meyer-Olkin (KMO) test and Bartlett’s test of sphericity. In addition, variance inflation factors (VIF) were computed to assess potential multicollinearity, while McDonald’s ω coefficients were examined to evaluate internal consistency reliability alongside other indicators. Subsequently, confirmatory factor analysis (CFA) was performed using maximum likelihood estimation (MLE) to further evaluate the measurement model’s goodness-of-fit. Model fit was comprehensively assessed through multiple indices, including the Goodness-of-Fit Index (GFI), Adjusted Goodness-of-Fit Index (AGFI), Normed Fit Index (NFI), Incremental Fit Index (IFI), Tucker-Lewis Index (TLI), Comparative Fit Index (CFI), Relative Fit Index (RFI), and Root Mean Square Error of Approximation (RMSEA). Second, internal consistency was examined using Cronbach’s α coefficient, while convergent validity was assessed through composite reliability (CR) and average variance extracted (AVE) [75]. Additionally, Harman’s single-factor test was employed to check for common method bias (CMB) by verifying whether the variance explained by the first factor was below the empirical threshold of 40% [76]. Third, in the SEM analysis, the bootstrap resampling method with 5000 subsamples was used to calculate standardized path coefficients and their significance levels for testing direct and mediating effects. The moderating role of gender in the pathways between behavioral intention and both self-efficacy and social influence was further examined using SPSS’s PROCESS macro (Model 7), systematically evaluating the statistical significance and directionality of each path relationship.

## Results

### Exploratory factor analysis

To ensure the validity of subsequent EFA, CFA, and SEM analyses, preliminary data diagnostics were conducted. Normality was assessed using skewness and kurtosis indices, all of which fell within the acceptable range (|skewness| < 2, |kurtosis| < 7), indicating no significant departure from normal distribution [77]. In addition, Mardia’s multivariate kurtosis coefficient was C.R. = 6.980, which exceeds the conventional threshold of 5.0 but remains below the flexible cutoff of 10 recommended for large samples (n > 200) [78,79], indicating acceptable multivariate normality for SEM. Boxplot inspection revealed no extreme outliers, which is consistent with the discrete nature of Likert-scale data. All missing responses were excluded during data cleaning, resulting in a complete dataset of 479 valid cases. Additionally, VIF values for all latent constructs were below 3.0, confirming the absence of multicollinearity issues [74]. These results suggest the dataset meets the assumptions required for robust multivariate analysis.

EFA was conducted using the Principal Component Analysis (PCA) method combined with varimax rotation. PCA, a widely adopted extraction technique, aims to reduce data dimensionality while retaining as much variance as possible in the dataset [80]. To facilitate interpretability, the factor solution was subjected to varimax rotation, an orthogonal method designed to maximize the variance of squared loadings within each factor, thereby producing a more distinct factor structure [81]. Based on the eigenvalue-greater-than-one criterion, the four factors respectively explained 30.595%, 14.552%, 11.347%, and 11.155% of the variance, and together accounted for 67.649% of the total variance, indicating a satisfactory factor structure. As shown in Table 2, all measured items exhibited factor loadings greater than 0.7, indicating that each item in the measurement model met statistical requirements and confirming good convergent validity of the research model. Second, internal consistency reliability was evaluated using Cronbach’s alpha, CR, and McDonald’s ω. Results showed that all latent variables had Cronbach’s alpha values ranging from 0.820 to 0.931 and CR values ranging from 0.821 to 0.931, all exceeding the recommended threshold of 0.7. McDonald’s ω values were also high, with 0.899 for BI, 0.893 for SE, 0.908 for SI, and 0.942 for TDC, further confirming the strong internal consistency of the measurement scales. Third, the AVE values for each latent variable ranged from 0.575 to 0.623, surpassing the critical value of 0.5, demonstrating that over 50% of the variance could be explained by the constructs and further supporting the measurement model’s adequate convergent validity. Additionally, Harman’s single-factor test was conducted to examine potential common method bias. The results extracted four factors with eigenvalues greater than 1, with the first factor explaining only 38.07% of the variance, below the 40% threshold, indicating no severe common method bias in this study [76].

**Table 2:** Measurement model validity and reliability.

Construct	Items	Loading	VIF	Cronbach’s α	McDonald’s ω	CR	AVE
BI	BI_1	0.766	1.984	0.831	0.899	0.832	0.623
	BI_2	0.776	1.986				
	BI_3	0.825	2.149				
SE	SE_1	0.801	2.102	0.820	0.893	0.821	0.604
	SE_2	0.759	2.110				
	SE_3	0.770	2.125				
SI	SI_1	0.778	2.301	0.864	0.908	0.864	0.615
	SI_2	0.773	2.021				
	SI_3	0.788	1.913				
	SI_4	0.797	1.914				
TDC	TDC_1	0.752	2.173	0.931	0.942	0.931	0.575
	TDC_2	0.746	2.143				
	TDC_3	0.766	2.231				
	TDC_4	0.774	2.283				
	TDC_5	0.770	2.338				
	TDC_6	0.753	2.170				
	TDC_7	0.753	2.223				
	TDC_8	0.774	2.303				
	TDC_9	0.744	2.120				
	TDC_10	0.750	2.163				

Note. BI: Behavioral intention; SE: Self-efficacy; SI: Social influence; TDC: Teachers’ digital competence.

### Confirmatory factor analysis

**Fig 2:**
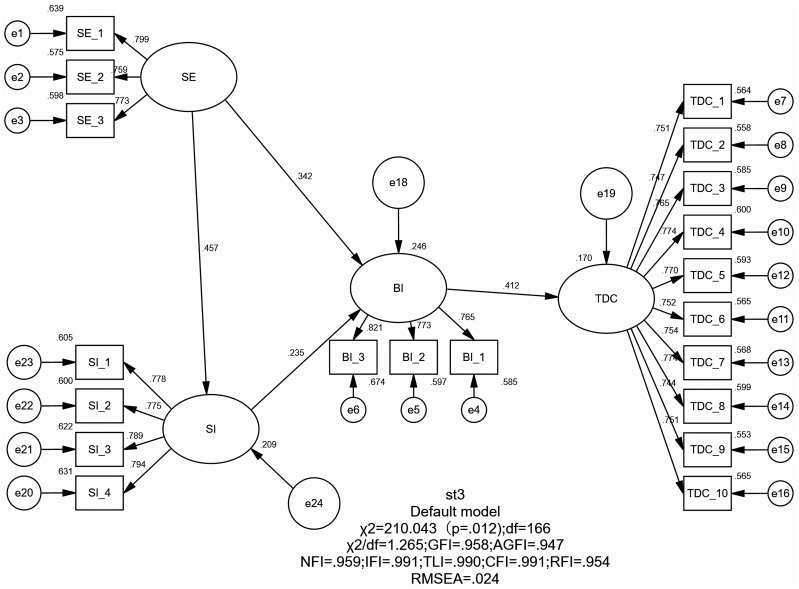
Classical theoretical structural equation model (M1) analysis.

**Fig 3:**
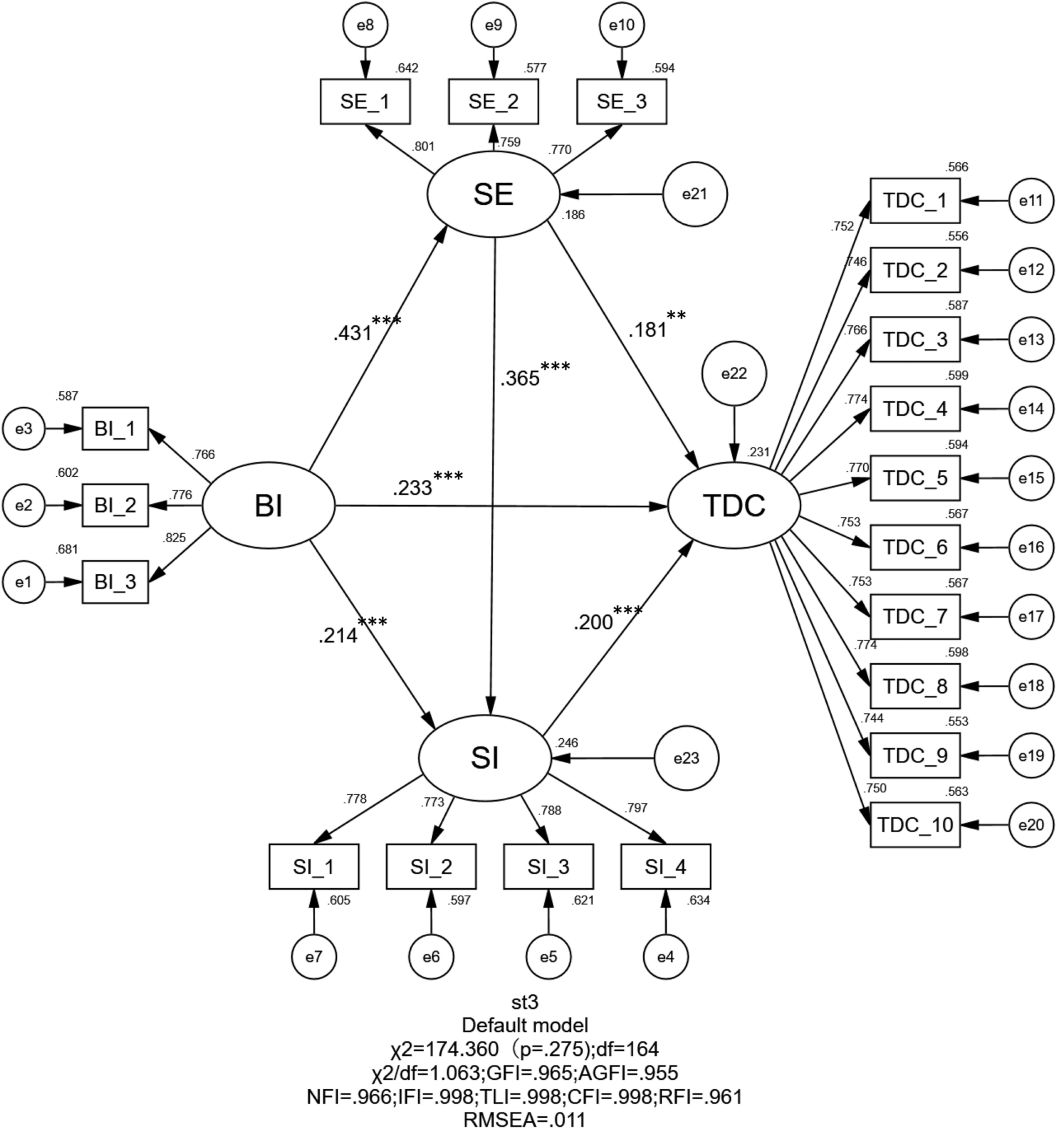
Proposed structural equation model (M2) analysis. Note. **p < .01, ***p < .001.

To evaluate the model fit and generalizability of the proposed pathway structure, this study conducted CFA comparing the traditional theoretical model with the proposed structural model. (a) The traditional theoretical model, referred to as M1 (see Fig 2), conceptualizes SE and SI as independent variables, BI as a mediating variable, and TDC as the dependent variable. (b) The proposed path model in this study, labeled as M2 (see Fig 3), positions BI as the independent variable, with SI and SE serving as mediators, and TDC as the dependent variable. Specifically, the comparative analysis of model fit indices was conducted to demonstrate that the path model linking college physical education teachers’ behavioral intention to use AI with their digital competence outperforms the traditional theoretical framework. The result shows the traditional path model (M1) yielded the following fit indices: GFI = 0.958, AGFI = 0.947, NFI = 0.959, CFI = 0.991, RMSEA = 0.024, and SRMR = 0.074. In contrast, the proposed model (M2) demonstrated superior fit indices: GFI = 0.965, AGFI = 0.955, NFI = 0.966, CFI = 0.998, RMSEA = 0.011, and SRMR = 0.028 (see Table 3). All indices for both models fall within the commonly accepted thresholds for good model fit [82]. The comparison of fit indices between M1 and M2, however, reveals that the proposed path model (M2) demonstrates superior performance over the traditional model (M1) in key metrics such as CFI, RMSEA, and SRMR. This suggests that, compared to the traditional model which treats BI as a mediating variable, the reverse specification of BI as an exogenous predictor in the present study provides a theoretically more compelling explanation.

**Table 3:** Comparative fit indices of the traditional and proposed path models.

Model	DF	X^2^	X^2^/df	GFI	AGFI	NFI	CFI	RMSEA	SRMR
M1	166.000	210.043	1.265	0.958	0.947	0.959	0.991	0.024	0.074
M2	164.000	174.360	1.063	0.965	0.955	0.966	0.998	0.011	0.028

### Direct path effects

As shown in Table 4, all direct hypotheses (H1–H6) were supported by the data, demonstrating strong model stability. Specifically, BI had a significant positive effect on SE (β = 0.431, t = 7.722, *p* < 0.001), supporting H1. This indicates that higher behavioral intention to use AI among university PE teachers enhances their self-efficacy in AI-assisted teaching. For H2, BI also positively influenced SI (β = 0.214, t = 3.744, *p* < 0.001), suggesting that stronger AI adoption intentions increase perceived social norms and peer/organizational support. In H6, SE significantly predicted SI (β = 0.365, t = 6.129, *p* < 0.001), implying that greater self-confidence in AI competence strengthens perceived social encouragement and normative pressure. Additionally, the analysis of digital competence (TDC) revealed that SE (β = 0.181, t = 3.029, *p* = 0.002), SI (β = 0.200, t = 3.569, *p* < 0.001), and BI (β = 0.233, t = 4.137, *p* < 0.001) all exerted significant positive effects on TDC, supporting H3, H4, and H5. These findings demonstrate that university PE teachers’ digital competence is driven not only by behavioral intention but also jointly shaped by self-efficacy and social influence.

**Table 4:** Path coefficient results (H1–H6).

H	Relationships	Standardized path (β)	T-value	*p*-value	Results
H1	BI → SE	0.431	7.722	<.001	Accepted
H2	BI → SI	0.214	3.744	<.001	Accepted
H3	SE → TDC	0.181	3.029	0.002	Accepted
H4	SI → TDC	0.200	3.569	<.001	Accepted
H5	BI → TDC	0.233	4.137	<.001	Accepted
H6	SE → SI	0.365	6.129	<.001	Accepted

Note. BI: Behavioral intention; SE: Self-efficacy; SI: Social influence; TDC: Teachers’ digital competence.

### Mediation and moderation effects

Building upon the latent variable modeling and CFA validation of the structural equation model (SEM), this study adopted an observed variable modeling strategy to further examine the mediating pathways and moderating mechanisms between BI and TDC. Following the “two-step modeling approach” [77], confirmatory factor analysis (CFA) was first conducted in AMOS to validate the measurement model, confirming good construct validity. Additionally, the squared multiple correlations (R^2^) from the SEM results indicated that the model explained 18.6% of the variance in SE, 24.6% in social influence (SI), and 23.1% in TDC, supporting the structural paths’ explanatory power and providing a solid foundation for subsequent analysis using observed variables. Subsequently, the mean values of each latent variable were extracted as observed variables and input into SPSS’s PROCESS v3.5 macro (Model 7) for analysis. In this model, BI was specified as the independent variable, TDC as the dependent variable, SI and SE as two parallel mediators, and Gender as the moderator affecting the paths from BI to the mediators (M1: SI and M2: SE). A 5000-sample bootstrap resampling method was employed to estimate path coefficients and confidence intervals (CI) at a 95% confidence level, with results presented in Table 5.

**Table 5:** Model coefficients for the conditional process analysis (PROCESS Model 7).

Antecedents				Consequent								
	M1 (SE)				M2 (SI)				Y (TDC)			
	Coeff.	StdError	p	95% CI	Coeff.	StdError	p	95% CI	Coeff.	StdError	p	95% CI
Constant	4.364	0.059	<.001	[4.248, 4.481]	4.371	0.06	<.001	[4.253, 4.490]	2.985	0.219	<.001	[2.556, 3.415]
BI	0.353	0.042	<.001	[0.270, 0.435]	0.311	0.043	<.001	[0.227, 0.395]	0.185	0.04	<.001	[0.107, 0.263]
Gender	–0.033	0.119	0.784	[-0.266, 0.201]	0.064	0.121	0.598	[-0.174, 0.302]				
BI × Gender	–0.043	0.084	0.605	[-0.208, 0.121]	0.011	0.086	0.896	[-0.157, 0.179]				
SE									0.156	0.042	<.001	[0.074, 0.238]
SI									0.176	0.041	<.001	[0.095, 0.256]
	R^2^ = 0.131, F = 23.786, *p* < .001				R^2^ = 0.102, F = 17.899, *p* < .001				R^2^ = 0.192, F = 37.545, *p* < .001			

Note. StdError: Standard error; BI: Behavioral intention; SE: Self-efficacy; SI: Social influence; TDC: Teachers’ digital competence.

The regression models demonstrated satisfactory explanatory power across all pathways. BI showed significant predictive effects on both SI (R^2^ = 0.102, F = 17.899, *p* < .001) and SE (R^2^ = 0.131, F = 23.786, *p* < .001), while the final TDC prediction model achieved R^2^ = 0.192 (F = 37.545, *p* < .001), indicating that BI, SI, and SE collectively explained significant variance in TDC. The R^2^ results from both AMOS and SPSS PROCESS jointly support the model’s structural stability and explanatory validity, reinforcing the central roles of BI, SE, and SI in accounting for variations in TDC within AI-supported physical education contexts. In addition, results (Table 6) revealed a direct effect of BI on TDC (β = 0.185, *p* < .001), with significant indirect effects through SE (β = 0.056, BootSE = 0.017, 95% CI [0.026, 0.090]) and SI (β = 0.055, BootSE = 0.017, 95% CI [0.025, 0.090]), confirming the existence of stable parallel mediation effects and supporting H7 and H8.

**Table 6:** Results of mediation and moderated mediation analyses (H7–H10).

H	Relationships	Effect (b)	BootSE	95% CI	*p*-value	Results	Proportion of effect
H7	Indirect (BI → SE → TDC)	0.056	0.016	[0.026, 0.090]	<.001	Accepted	18.90%
H8	Indirect (BI → SI → TDC)	0.055	0.017	[0.025, 0.090]	<.001	Accepted	18.60%
H9	Gender moderates (BI → SE)	–0.043	0.084	[–0.208, 0.121]	0.605	Rejected	
H10	Gender moderates (BI → SI)	0.011	0.086	[–0.157, 0.179]	0.896	Rejected	
	Direct effect (BI → TDC)	0.185	0.040	[0.107, 0.263]	<.001		62.50%
	Total indirect mediation effect	0.111					37.50%
	Total mediation effect	0.296					

Note. b = Unstandardized coefficient; BootSE = Bootstrap standard error. BI: Behavioral intention; SE: Self-efficacy; SI: Social influence; TDC: Teachers’ digital competence.

Regarding gender moderation, the interaction terms in the mediation pathways showed non-significant effects for both BI × Gender on SI (b = 0.011, *p* = 0.896) and on SE (b = –0.043, *p* = 0.605), indicating no significant moderating role of gender in the first-stage mediation paths. Thus, H9 and H10 were not supported.

In summary, the findings validate robust indirect pathways through which university physical education teachers’ BI influences TDC via SI and SE, advancing the integrated understanding of UTAUT and SCT in technology adoption contexts.

## Discussion

This study employed a questionnaire survey based on the UTAUT model and SCT theory to investigate the relationship between university physical education teachers’ behavioral intention to use AI and their digital competence. We developed a structural model including behavioral intention, self-efficacy, social influence, and teachers’ digital competence, and proposed ten research hypotheses tested through data analysis.

### Direct effects of behavioral intention on self-efficacy, social influence, and teacher digital competence

The findings demonstrate that BI plays a pivotal driving role in university physical education teachers’ AI adoption pathways. First, BI is significantly and positively related to SE, indicating that when physical education teachers develop strong intentions to use AI, it reflects cognitive preparation and mobilization, thereby enhancing their perceived capability to execute the task. Although mainstream research typically treats SE as an antecedent of BI [22,47], this study reveals that in the context of AI adoption in education, forming clear behavioral intentions may, through cognitive activation and situational expectations, be associated with greater self-perceived competence. This challenges the unidirectional causality in traditional SCT and warrants further longitudinal research to explore its underlying mechanisms and boundary conditions.

Second, BI is also significantly connected with SI, suggesting that once teachers establish firm usage intentions, they become more receptive to positive organizational evaluations of AI technology, highlighting the proactive interplay between individual agencies and external environments. Although SI is commonly viewed as an antecedent of BI, emerging studies, such as Ishaq et al. [30], propose that technology adoption behaviors may reciprocally be related to user beliefs, supporting the existence of feedback mechanisms in technology acceptance. This aligns with and substantiates the potential role of physical education teachers’ AI usage intentions in influencing perceived social norms.

Furthermore, BI is directly and significantly associated with TDC, implying that with clear AI adoption intentions, PE teachers are more likely to proactively explore, learn, and integrate relevant technologies, thereby being related to improved digital capabilities [83]. Concurrently, SE shows a positive relationship with TDC, underscoring that highly efficacious teachers are more willing to experiment with AI and invest in skill development [84]. The positive association of SI with TDC further validates the importance of external support, as organizational endorsement fosters competency building. De la Calle et al. [85] emphasize that teachers’ digital competence benefits not only students but also community social cohesion and quality of life, underscoring the importance of sociocultural factors in competency development.

Additionally, SE is significantly related to SI, revealing a critical psychological mechanism: when teachers believe in their technical abilities, they are more likely to perceive organizational climates as supportive, creating a synergistic reinforcement between internal and external motivators. This finding aligns with Sung et al. [86], who reported that higher self-efficacy is associated with stronger perceived social influence. Collectively, these results demonstrate that PE teachers’ self-efficacy and social influence jointly contribute to their digital competence advancement.

### Mediating effects of self-efficacy and social influence

This study reveals that BI is indirectly associated with university physical education teachers’ digital competence through SE, suggesting a robust internal psychological mechanism. From the perspective of social cognitive theory, Bandura [87] posits that mastering new technologies and gaining successful experiences most effectively strengthen self-efficacy, aligning with our findings. Teachers willing to adopt AI actively engage with the technology, and their confidence strengthens upon successful mastery, thereby being linked to digital competence improvement. A Chinese study corroborates this, showing that higher self-efficacy among university teachers is significantly related to their digital capabilities in technological contexts [84]. Thus, PE teachers’ AI usage intention is associated with digital competence by reinforcing self-efficacy, with this mediation mechanism playing a pivotal role.

Simultaneously, BI is significantly related to TDC through SI, forming an externally motivated pathway. Teachers with stronger AI adoption intentions actively participate in technology-oriented teaching communities, perceiving and internalizing support and validation from peers, leaders, and institutional environments, which further motivates their AI learning and usage. Relevant studies indicate that teachers in supportive social influence contexts more readily adopt and integrate digital technologies into their teaching practices [88]. Additionally, Jere and Mpeta [89] found that supportive social norms in organizational culture are critical for facilitating teachers’ technology integration. Consequently, this study uncovers a less-explored dual mediation structure (psychological and social), showing that PE teachers’ AI usage intention is associated with higher digital competence both through internalized SE and externalized SI. This emphasis on indirect pathways aligns with evidence that the SMART program improves students’ cognitive, academic, personal, and social outcomes, which collectively shape career decisions rather than exerting direct effects [90].

It is worth emphasizing that, although the proposed path model in this study demonstrates a high level of statistical fit, this does not necessarily indicate model overfitting or a saturated structural configuration. On the one hand, the model structure is derived from the integrative logic of SCT and UTAUT, with theoretically grounded path specifications that avoid empirically driven path proliferation, thereby retaining essential degrees of freedom and ensuring theoretical rigor. On the other hand, to ensure the robustness and credibility of the model results, we implemented rigorous procedures throughout the questionnaire design and administration process. Specifically, the development of the measurement instruments was carefully contextualized to reflect the actual working scenarios of university physical education teachers, ensuring a high degree of relevance and applicability to the target population. During the questionnaire administration phase, the research team implemented a combination of online and offline supervision strategies to monitor and screen responses. Following data collection, rigorous data cleaning and validation procedures were carried out in accordance with established statistical standards to ensure data integrity. I addition, we conducted robustness verification of the path coefficients using Bootstrap resampling (n = 5000). The confidence intervals of key paths did not cross zero, indicating that the relationships were both statistically significant and stable. In summary, the study indicates methodological rigor and theoretical coherence in both model construction and empirical implementation, thereby minimizing the risk of model overfitting.

### Explanation of non-significant moderation

The study found that gender did not significantly moderate the relationship between university physical education teachers’ BI and either SE or SI. Recent research suggests the moderating effect of gender among higher education faculty is diminishing, particularly as teaching roles become more professionalized and digital skills more ubiquitous. For instance, Konukman and Filiz’s [91] study of Turkish PE teachers similarly found no significant gender differences in technology innovation adoption and diffusion.

Notably, university PE teachers, as practice-oriented educators with strong contextual adaptability, appear more related to pedagogical needs and organizational culture than by gender when adopting technology. Research indicates that in contexts of rapid technological advancement and educational digital transformation, teachers’ technology adoption is more closely associated with perceived usefulness and institutional support [92]. This is further supported by Amoako and Anane’s [93] Ghanaian study, which found no significant gender moderation between digital teaching competence and teacher resilience.

These findings collectively suggest a gender-neutral trend in university physical education teachers’ AI adoption intentions, with gender no longer serving as a critical factor in digital competence development. The results align with broader patterns of decreasing gender disparities in technology adoption among education professionals. Nevertheless, given the cross-sectional design, these relationships should be interpreted as correlational rather than causal. Future longitudinal or experimental research is required to determine the temporal and causal sequence of these associations, thereby providing more definitive evidence regarding the underlying mechanisms for teachers’ adoption of artificial intelligence and the development of digital competencies.

## Conclusion

### Summary of key findings

This study employs an integrated framework combining SCT and UTAUT to examine the relationship between university physical education teachers’ behavioral intention to use AI and their digital competence. The findings indicate that physical education teachers’ BI significantly and positively predicts their SE, perceived SI, and TDC, indicating that those with stronger AI adoption intentions exhibit greater intrinsic motivation and external connectivity awareness, thereby being linked to higher digital competence. SE and SI function as strong mediators between BI and TDC, forming a dual “psychological–social” pathway in which internal and external factors work together. Furthermore, SE amplifies teachers’ sensitivity to social support, creating a mutually reinforcing mechanism. Moderating effect analysis reveals that gender does not show a significant association with the relationships between BI and SE/SI, suggesting gender-neutral patterns in AI adoption pathways within higher education physical education contexts. Collectively, this research elucidates the mechanisms linking physical education teachers’ AI usage intentions with digital competence development, providing theoretical foundations and practical insights for advancing digital transformation in physical education pedagogy. The results highlight the importance of fostering both psychological empowerment and social-environmental support to facilitate technology integration in specialized educational settings.

### Theoretical and practical implications

This study makes three primary theoretical contributions. First, this study constructed a structural model integrating SCT and UTAUT, providing a more comprehensive theoretical framework for systematically understanding the correlation mechanism between college physical education teachers’ willingness to use AI and their digital competence. Unlike traditional models that treat self-efficacy and social influence as predictors of behavioral intention, this study reverses the logic by positioning behavioral intention as an independent variable to examine its mediating effects on self-efficacy and social influence. This novel approach helps clarify the link between teachers’ AI usage intention and their digital competence. Furthermore, it effectively expands the theoretical scope of SCT and UTAUT in educational technology research, particularly demonstrating good theoretical adaptability and model explanatory power in the specific group of college physical education teachers. Second, this study further clarified the indirect causal pathways between self-efficacy, social influence, and digital competence, enriching our understanding of the mediating mechanisms in educational technology adoption behavior. This indicates that AI usage intention can indirectly be related to technical capabilities by strengthening individual beliefs and social recognition, further expanding the mediating role of self-efficacy and social influence in explaining physical education teachers’ AI usage intention. Third, this study empirically tested the moderating effect of gender. The results showed that gender did not have a significant effect on the path from behavioral intention to SE and SI. This finding provides theoretical inspiration for re-examining demographic variables in studies related to teachers’ adoption of AI technology.

At the practical level, the study offers practical implications for promoting AI integration in physical education. First, universities need to establish a stable and feasible institutional support system to promote teachers’ adoption of AI, enhancing their initiative and enthusiasm in using AI in teaching and education through infrastructure improvements and policy implementation. Second, universities should enhance physical education teachers’ self-efficacy in using AI through a structured, progressive training system. This involves designing tiered training programs tailored to teachers’ job roles, skill levels, and instructional responsibilities. For example, combining virtual simulation classrooms, AI tool application training, and peer review mechanisms can continuously strengthen teachers’ confidence and competence in using AI in the teaching process. Third, universities should enhance teachers’ understanding of AI usage by establishing AI teaching demonstration positions, organizing interdisciplinary teaching and research collaboration groups, and promoting open class exchanges among teachers. This will facilitate technical transfer and mutual learning among teachers in the use of AI. Fourth, universities should incorporate AI technology into their overall strategy for innovating sports course content and restructuring teaching models. They should establish AI-based intelligent sports assessment platforms, build dynamic student feedback mechanisms, and promote personalized adjustments to teaching objectives. These measures will contribute to the effectiveness of AI technology in education and teaching, and help physical education teachers achieve digital transformation.

### Study limitations and future prospects

While this study establishes a theoretically grounded and methodologically robust analytical framework, yielding systematic findings on the mechanisms linking university physical education teachers’ AI usage intentions with digital competence, several limitations warrant attention in future research. First, this study integrates SCT and UTAUT to provide a new research perspective for understanding the mechanism relationship between college physical education teachers’ willingness to use AI and their digital competence. But this integration approach can be further enriched in future research by incorporating the Innovation Diffusion Theory (IDT) and the Technology-Organization-Environment (TOE) framework, thereby contributing to the multidimensionality of theoretical construction and expanding the explanatory power of AI technology adoption behavior in the education sector. Second, though the sample represents Chinese mainland university physical education teachers, generalizability remains constrained by regional cultural and institutional contexts. Comparative studies across diverse national education systems would help strengthen the universality and external validity of the findings. Third, the quantitative approach, while validating pathway relationships, lacks depth in capturing teachers’ subjective experiences and behavioral logics in real-world AI implementation. Mixed-methods designs integrating interviews or classroom observations could provide richer, more holistic insights. Lastly, although this study theoretically constructed the structural path between college physical education teachers’ willingness to use AI and their digital competence, and revealed the mediating role of self-efficacy and social influence, there is still room for improvement in terms of further expansion in specific practical dimensions. Future research could incorporate action research or intervention-based research approaches, integrating variables such as educational management policies, resource allocation efficiency, and technical support conditions. This would enable a systemic exploration of diverse strategies that may support the AI adoption capabilities of university physical education teachers, thereby facilitating the effective translation of theoretical models into educational practice. Addressing these limitations would strengthen both theoretical and practical contributions to understanding technology adoption in specialized educational contexts.

## Supporting information

S1 Table Measurement items (in English and Chinese language) adopted. (DOCX)

## References

[1] Hazzan-Bishara A, Kol O, Levy S. The factors affecting teachers’ adoption of AI technologies: a unified model of external and internal determinants. Educ Inf Technol. 2025;30(11):15043–69. doi: 10.1007/s10639-025-13393-z

[2] Surveys: Educators Approve of ChatGPT for K-12, College. Government Technology. https://www.govtech.com/education/k-12/survey-educators-approve-of-chatgpt-for-k-12-college. 2023. Accessed 2025 May 3.

[3] Kim NJ, Kim MK. Teacher’s Perceptions of Using an Artificial Intelligence-Based Educational Tool for Scientific Writing. Front Educ. 2022;7. doi: 10.3389/feduc.2022.755914

[4] Nevárez Montes J, Elizondo-Garcia J. Faculty acceptance and use of generative artificial intelligence in their practice. Front Educ. 2025;10. doi: 10.3389/feduc.2025.1427450

[5] Kyrpa A, Stepanenko O, Zinchenko V, Datsiuk T, Karpan I, Tilniak N. Artificial intelligence tools in teaching social and humanitarian disciplines. ITLT. 2024;100(2):162–79. doi: 10.33407/itlt.v100i2.5563

[6] Molefi RR, Ayanwale MA, Kurata L, Chere-Masopha J. Do in-service teachers accept artificial intelligence-driven technology? The mediating role of school support and resources. Computers and Education Open. 2024;6:100191. doi: 10.1016/j.caeo.2024.100191

[7] Chen X, Hu Z, Wang C. Empowering education development through AIGC: a systematic literature review. Educ Inf Technol. 2024;29(13):17485–537. doi: 10.1007/s10639-024-12549-7

[8] Wu W, Zhang B, Li S, Liu H. Exploring factors of the willingness to accept ai-assisted learning environments: an empirical investigation based on the UTAUT model and perceived risk theory. Front Psychol. 2022;13:870777. doi: 10.3389/fpsyg.2022.870777 35814061 PMC9270016

[9] Chatterjee S, Bhattacharjee KK. Adoption of artificial intelligence in higher education: a quantitative analysis using structural equation modelling. Educ Inf Technol. 2020;25(5):3443–63. doi: 10.1007/s10639-020-10159-7

[10] Brands C, Mayer C-H, Oosthuizen RM. Chartered Accountants’ perception of the Fourth Industrial Revolution. Front Psychol. 2024;15:1419766. doi: 10.3389/fpsyg.2024.1419766 39483403 PMC11524987

[11] Wang J, Zhou Y, Tan K, Yu Z, Li Y. Acceptance of artificial intelligence clinical assistant decision support system to prevent and control venous thromboembolism among healthcare workers: an extend Unified Theory of Acceptance and Use of Technology Model. Front Med (Lausanne). 2025;12:1475577. doi: 10.3389/fmed.2025.1475577 40007590 PMC11850527

[12] Ateş H, Gündüzalp C. Proposing a conceptual model for the adoption of artificial intelligence by teachers in STEM education. Interactive Learning Environments. 2025;33(6):4020–46. doi: 10.1080/10494820.2025.2457350

[13] Chiu TKF, Falloon G, Song Y, Wong VWL, Zhao L, Ismailov M. A self-determination theory approach to teacher digital competence development. Computers & Education. 2024;214:105017. doi: 10.1016/j.compedu.2024.105017

[14] Falloon G. From digital literacy to digital competence: the teacher digital competency (TDC) framework. Education Tech Research Dev. 2020;68(5):2449–72. doi: 10.1007/s11423-020-09767-4

[15] Ng DTK, Leung JKL, Su J, Ng RCW, Chu SKW. Teachers’ AI digital competencies and twenty-first century skills in the post-pandemic world. Educ Technol Res Dev. 2023;71(1):137–61. doi: 10.1007/s11423-023-10203-6 36844361 PMC9943036

[16] Zhou T, Wu X, Wang Y, Wang Y, Zhang S. Application of artificial intelligence in physical education: a systematic review. Educ Inf Technol. 2023;29(7):8203–20. doi: 10.1007/s10639-023-12128-2

[17] GENÇ N. Artificial Intelligence in Physical Education and Sports: New Horizons with ChatGPT. MJSS. 2023. doi: 10.38021/asbid.1291604

[18] An R, Shen J, Wang J, Yang Y. A scoping review of methodologies for applying artificial intelligence to physical activity interventions. J Sport Health Sci. 2024;13(3):428–41. doi: 10.1016/j.jshs.2023.09.010 37777066 PMC11116969

[19] Vidot DC, Rethorst CD, Carmody TJ, Stoutenberg M, Walker R, Greer TL, et al. Acute and long-term cannabis use among stimulant users: Results from CTN-0037 Stimulant Reduction Intervention using Dosed Exercise (STRIDE) Randomized Control Trial. Drug Alcohol Depend. 2019;200:139–44. doi: 10.1016/j.drugalcdep.2019.02.032 31129484 PMC6863445

[20] Rashid AB, Kausik MAK. AI revolutionizing industries worldwide: A comprehensive overview of its diverse applications. Hybrid Advances. 2024;7:100277. doi: 10.1016/j.hybadv.2024.100277

[21] Bandura A. Social foundations of thought and action: A social cognitive theory. Englewood Cliffs, NJ: Prentice-Hall, Inc. 1986.

[22] Venkatesh, Morris, Davis, Davis. User Acceptance of Information Technology: Toward a Unified View. MIS Quarterly. 2003;27(3):425. doi: 10.2307/30036540

[23] Li X, Zhang J, Yang J. The effect of computer self-efficacy on the behavioral intention to use translation technologies among college students: Mediating role of learning motivation and cognitive engagement. Acta Psychol (Amst). 2024;246:104259. doi: 10.1016/j.actpsy.2024.104259 38608364

[24] Bandura A, Freeman WH, Lightsey R. Self-efficacy: the exercise of control. J Cogn Psychother. 1999;13(2):158–66. doi: 10.1891/0889-8391.13.2.158

[25] Schunk DH, DiBenedetto MK. Motivation and social cognitive theory. Contemporary Educational Psychology. 2020;60:101832. doi: 10.1016/j.cedpsych.2019.101832

[26] Li S, Xu R, Zhao Z. Innovation in physical education: The role of cognitive factors and self-efficacy. Front Psychol. 2022;13:959979. doi: 10.3389/fpsyg.2022.959979 36033041 PMC9399811

[27] Lin JB, Zhu SS. The influencing factors of individual interest in physical education based on decision tree model: A cross-sectional study. Front Psychol. 2022;13:1015441. doi: 10.3389/fpsyg.2022.1015441 36300076 PMC9589482

[28] Casey A, Goodyear VA, Armour KM. Rethinking the relationship between pedagogy, technology and learning in health and physical education. Sport, Education and Society. 2016;22(2):288–304. doi: 10.1080/13573322.2016.1226792

[29] Ma L, Chee CS, Amri S, Gao X, Wang Q, Wang N, et al. Impact of self-efficacy and burnout on professional development of physical education teachers in the digital age: a systematic review. PeerJ. 2025;13:e18952. doi: 10.7717/peerj.18952 39995991 PMC11849512

[30] Ishaq E, Bashir S, Zakariya R, Sarwar A. Technology acceptance behavior and feedback loop: exploring reverse causality of TAM in Post-COVID-19 scenario. Front Psychol. 2021;12:682507. doi: 10.3389/fpsyg.2021.682507 34589017 PMC8473738

[31] Conger JA, Kanungo RN. The Empowerment Process: Integrating Theory and Practice. The Academy of Management Review. 1988;13(3):471. doi: 10.2307/258093

[32] Gollwitzer PM. Implementation intentions: Strong effects of simple plans. Am Psychologist. 1999;54(7):493–503. doi: 10.1037/0003-066x.54.7.493

[33] Schwarzer R, Renner B. Social-cognitive predictors of health behavior: Action self-efficacy and coping self-efficacy. Health Psychology. 2000;19(5):487–95. doi: 10.1037/0278-6133.19.5.487 11007157

[34] Xue L, Rashid AM, Ouyang S. The Unified Theory of Acceptance and Use of Technology (UTAUT) in Higher Education: A Systematic Review. Sage Open. 2024;14(1). doi: 10.1177/21582440241229570

[35] Philippi P, Baumeister H, Apolinário-Hagen J, Ebert DD, Hennemann S, Kott L, et al. Acceptance towards digital health interventions - Model validation and further development of the Unified Theory of Acceptance and Use of Technology. Internet Interv. 2021;26:100459. doi: 10.1016/j.invent.2021.100459 34603973 PMC8463857

[36] Katebi A, Tehrani M. Adoption of AI in construction design: Insights from UTAUT2 and TOE frameworks. Results in Engineering. 2025;26:104981. doi: 10.1016/j.rineng.2025.104981

[37] Zacharis G, Nikolopoulou K. Factors predicting university students’ behavioral intention to use eLearning platforms in the post-pandemic normal: an UTAUT2 approach with “Learning Value”. Educ Inf Technol (Dordr). 2022;27(9):12065–82. doi: 10.1007/s10639-022-11116-2 35645598 PMC9130973

[38] Chand SS, Kumar BA. Applying the UTAUT model to understand m-payment adoption. a case study of western part of fiji. J Knowl Econ. 2024;15(4):15523–49. doi: 10.1007/s13132-023-01722-x

[39] Compeau DR, Higgins CA. Computer self-efficacy: development of a measure and initial test. MIS Quarterly. 1995;19(2):189. doi: 10.2307/249688

[40] Graf-Vlachy L, Buhtz K, König A. Social influence in technology adoption: taking stock and moving forward. Manag Rev Q. 2018;68(1):37–76. doi: 10.1007/s11301-017-0133-3

[41] Caena F, Redecker C. Aligning teacher competence frameworks to 21st century challenges: The case for the European Digital Competence Framework for Educators (Digcompedu). Euro J of Education. 2019;54(3):356–69. doi: 10.1111/ejed.12345

[42] Smestad B, Hatlevik OE, Johannesen M, Øgrim L. Examining dimensions of teachers’ digital competence: A systematic review pre- and during COVID-19. Heliyon. 2023;9(6):e16677. doi: 10.1016/j.heliyon.2023.e16677 37292364 PMC10245064

[43] García-Delgado MÁ, Rodríguez-Cano S, Delgado-Benito V, Di Giusto-Valle C. Digital teaching competence among teachers of different educational stages in Spain. Education Sciences. 2023;13(6):581. doi: 10.3390/educsci13060581

[44] Vieira RM, Tenreiro-Vieira CC, Bem-Haja P, Lucas M. STEM teachers’ digital competence: different subjects, different proficiencies. Education Sciences. 2023;13(11):1133. doi: 10.3390/educsci13111133

[45] Lan H, Bailey R, Tan WH. Assessing the digital competence of in-service university educators in China: A systematic literature review. Heliyon. 2024;10(16):e35675. doi: 10.1016/j.heliyon.2024.e35675 39220952 PMC11365341

[46] Rojo-Ramos J, Carlos-Vivas J, Manzano-Redondo F, Fernández-Sánchez MR, Rodilla-Rojo J, García-Gordillo MÁ, et al. Study of the Digital Teaching Competence of Physical Education Teachers in Primary Schools in One Region of Spain. Int J Environ Res Public Health. 2020;17(23):8822. doi: 10.3390/ijerph17238822 33261068 PMC7730711

[47] Bandura A. Self-efficacy: The exercise of control. 1st ed. New York: Worth Publishers. 1997.

[48] Teo T. Modelling technology acceptance in education: A study of pre-service teachers. Computers & Education. 2009;52(2):302–12. doi: 10.1016/j.compedu.2008.08.006

[49] Elstad E, Christophersen K-A. Perceptions of digital competency among student teachers: contributing to the development of student teachers’ instructional self-efficacy in technology-rich classrooms. Education Sciences. 2017;7(1):27. doi: 10.3390/educsci7010027

[50] Buraimoh OF, Boor CHM, Aladesusi GA. Examining Facilitating Condition and Social Influence as Determinants of Secondary School Teachers’ Behavioural Intention to Use Mobile Technologies for Instruction. Indonesian J Educ Res Technol. 2022;3(1):25–34. doi: 10.17509/ijert.v3i1.44720

[51] Tondeur J, Aesaert K, Prestridge S, Consuegra E. A multilevel analysis of what matters in the training of pre-service teacher’s ICT competencies. Comput Education. 2018;122:32–42. doi: 10.1016/j.compedu.2018.03.002

[52] Instefjord E, Munthe E. Preparing pre-service teachers to integrate technology: an analysis of the emphasis on digital competence in teacher education curricula. European J Teacher Education. 2015;39(1):77–93. doi: 10.1080/02619768.2015.1100602

[53] Nazaretsky T, Ariely M, Cukurova M, Alexandron G. Teachers’ trust in AI‐powered educational technology and a professional development program to improve it. Brit J Educational Tech. 2022;53(4):914–31. doi: 10.1111/bjet.13232

[54] Stumbrienė D, Jevsikova T, Kontvainė V. Key factors influencing teachers’ motivation to transfer technology-enabled educational innovation. Educ Inf Technol (Dordr). 2023;:1–35. doi: 10.1007/s10639-023-11891-6 37361759 PMC10199451

[55] Scherer R, Siddiq F, Tondeur J. The technology acceptance model (TAM): A meta-analytic structural equation modeling approach to explaining teachers’ adoption of digital technology in education. Computers & Education. 2019;128:13–35. doi: 10.1016/j.compedu.2018.09.009

[56] Bayaga A. Leveraging AI-enhanced and emerging technologies for pedagogical innovations in higher education. Educ Inf Technol. 2024;30(1):1045–72. doi: 10.1007/s10639-024-13122-y

[57] Mahmood A, Huang X, Rehman N. STEM education as a catalyst for career aspirations and 21st‐century competences: insights from teachers’ perspectives. School Sci & Mathematics. 2025. doi: 10.1111/ssm.18381

[58] Teo T, Sang G, Mei B, Hoi CKW. Investigating pre-service teachers’ acceptance of Web 2.0 technologies in their future teaching: a Chinese perspective. Interactive Learning Environments. 2018;27(4):530–46. doi: 10.1080/10494820.2018.1489290

[59] Basilotta-Gómez-Pablos V, Matarranz M, Casado-Aranda L-A, Otto A. Teachers’ digital competencies in higher education: a systematic literature review. Int J Educ Technol High Educ. 2022;19(1). doi: 10.1186/s41239-021-00312-8

[60] Tondeur J, Scherer R, Siddiq F, Baran E. A comprehensive investigation of TPACK within pre-service teachers’ ICT profiles: Mind the gap!. AJET. 2017;33(3). doi: 10.14742/ajet.3504

[61] Vongkulluksn VW, Xie K, Bowman MA. The role of value on teachers’ internalization of external barriers and externalization of personal beliefs for classroom technology integration. Computers & Education. 2018;118:70–81. doi: 10.1016/j.compedu.2017.11.009

[62] An F, Xi L, Yu J, Zhang M. Relationship between technology acceptance and self-directed learning: mediation role of positive emotions and technological self-efficacy. Sustainability. 2022;14(16):10390. doi: 10.3390/su141610390

[63] Adams A, Belcher D. Physical Education Teachers’ Technology Self-Efficacy and Integration. TPE. 2025;82(3). doi: 10.18666/tpe-2025-v82-i3-11236

[64] Feng J, Yu B, Tan WH, Dai Z, Li Z. Key factors influencing educational technology adoption in higher education: A systematic review. PLOS Digit Health. 2025;4(4):e0000764. doi: 10.1371/journal.pdig.0000764 PMC12040101 40299977

[65] Huang H-C, Kung Y-T, Huang R-R, Mui W-C, Su Y-C. Assessment of physical education teachers’ use of distance teaching behavior under the influence of the COVID-19 pandemic. PeerJ. 2025;13:e18743. doi: 10.7717/peerj.18743 39850829 PMC11756359

[66] Saiz-González P, Sierra-Díaz J, Iglesias D, Fernandez-Rio J. Exploring physical education teachers’ willingness and barriers to integrating digital technology in their lessons. Educ Inf Technol. 2024;30(5):5965–87. doi: 10.1007/s10639-024-13060-9

[67] Bussey K, Bandura A. Social cognitive theory of gender development and differentiation. Psychol Rev. 1999;106(4):676–713. doi: 10.1037/0033-295x.106.4.676 10560326

[68] Xia Q, Chiu TKF, Chai CS. The moderating effects of gender and need satisfaction on self-regulated learning through Artificial Intelligence (AI). Educ Inf Technol. 2022;28(7):8691–713. doi: 10.1007/s10639-022-11547-x

[69] Scherer R, Siddiq F. Revisiting teachers’ computer self-efficacy: A differentiated view on gender differences. Computers in Human Behavior. 2015;53:48–57. doi: 10.1016/j.chb.2015.06.038

[70] Chao C-M. Factors Determining the Behavioral Intention to Use Mobile Learning: An Application and Extension of the UTAUT Model. Front Psychol. 2019;10:1652. doi: 10.3389/fpsyg.2019.01652 31379679 PMC6646805

[71] Wu Q, Li S, Xin S, Hou Q, Li P. A study on students’ behavioural intention and use behaviour of artificial intelligence-generated content in physical education: Employing an extended the unified theory of acceptance and use of technology model. J Hospitality Leisure Sport Tourism Education. 2025;36:100547. doi: 10.1016/j.jhlste.2025.100547

[72] Mieg HA, Klieme KE, Barker E, Bryan J, Gibson C, Haberstroh S, et al. Short digital-competence test based on DigComp2.1: Does digital competence support research competence in undergraduate students?. Educ Inf Technol. 2023;29(1):139–60. doi: 10.1007/s10639-023-12251-0

[73] Faul F, Erdfelder E, Buchner A, Lang A-G. Statistical power analyses using G*Power 3.1: tests for correlation and regression analyses. Behav Res Methods. 2009;41(4):1149–60. doi: 10.3758/BRM.41.4.1149 19897823

[74] Kline RB. Principles and practice of structural equation modeling. 5th ed. New York, NY: Guilford Press. 2023.

[75] Fornell C, Larcker DF. Evaluating Structural Equation Models with Unobservable Variables and Measurement Error. J Marketing Res. 1981;18(1):39–50. doi: 10.1177/002224378101800104

[76] Podsakoff PM, MacKenzie SB, Lee J-Y, Podsakoff NP. Common method biases in behavioral research: a critical review of the literature and recommended remedies. J Appl Psychol. 2003;88(5):879–903. doi: 10.1037/0021-9010.88.5.879 14516251

[77] Hair JF, Black WC, Babin BJ, Anderson RE. Multivariate data analysis. 7th ed. Upper Saddle River, NJ: Pearson Education. 2014.

[78] Collier J. Applied structural equation modeling using AMOS: Basic to advanced techniques. 1st ed. New York: Routledge. 2020.

[79] West SG, Finch JF, Curran PJ. Structural equation models with nonnormal variables: Problems and remedies. In: Hoyle RH, editor. Structural equation modeling: Concepts, issues, and applications. Thousand Oaks: Sage Publications, Inc. 1995. p. 56–75.

[80] Harman HH. Modern factor analysis. 3rd ed. Chicago: University of Chicago Press. 1976.

[81] Kaiser HF. The Varimax Criterion for Analytic Rotation in Factor Analysis. Psychometrika. 1958;23(3):187–200. doi: 10.1007/bf02289233

[82] Hu L, Bentler PM. Cutoff criteria for fit indexes in covariance structure analysis: Conventional criteria versus new alternatives. Structural Equation Modeling: A Multidisciplinary Journal. 1999;6(1):1–55. doi: 10.1080/10705519909540118

[83] Al-Rahmi WM, Yahaya N, Aldraiweesh AA, Alamri MM, Aljarboa NA, Alturki U, et al. Integrating technology acceptance model with innovation diffusion theory: an empirical investigation on students’ intention to Use E-learning systems. IEEE Access. 2019;7:26797–809. doi: 10.1109/access.2019.2899368

[84] Wang Z, Chu Z. Examination of higher education teachers’ self-perception of digital competence, self-efficacy, and facilitating conditions: an empirical study in the context of China. Sustainability. 2023;15(14):10945. doi: 10.3390/su151410945

[85] De la Calle AM, Pacheco-Costa A, Gómez-Ruiz MÁ, Guzmán-Simón F. Understanding teacher digital competence in the framework of social sustainability: a systematic review. Sustainability. 2021;13(23):13283. doi: 10.3390/su132313283

[86] Sung HN, Jeong DY, Jeong YS, Shin JI. The relationship among self-efficacy, social influence, performance expectancy, effort expectancy, and behavioral intention in mobile learning service. Int J u- e-Serv Sci Technol. 2015;8(9):197–206.

[87] Bandura A. Reconstrual of “free will” from the agentic perspective of social cognitive theory. In: Baer J, Kaufman JC, Baumeister RF, editors. Are we free? Psychology and free will. Oxford: Oxford University Press. 2008. p. 86–127.

[88] Yang L, García-Holgado A, Martínez-Abad F. Digital competence of K-12 pre-service and in-service teachers in China: a systematic literature review. Asia Pacific Educ Rev. 2023;24(4):679–93. doi: 10.1007/s12564-023-09888-4

[89] Jere S, Mpeta M. Effects of facilitating condition, social influence and self-efficacy on science teachers’ integration of digital technology in south africa: a regression-based approach. IJLTER. 2024;23(4):354–75. doi: 10.26803/ijlter.23.4.19

[90] Mahmood A, Rehman N, Huang X, Zamani N. Effect of strategic memory advanced reasoning training (SMART) therapy for enhancing final-year high school students career choices. BMC Psychol. 2025;13(1):445. doi: 10.1186/s40359-025-02767-0 40296108 PMC12036163

[91] Konukman F, Filiz B. Turkish Physical Education Teachers’ Use of Technology: Application and Diffusion of Technological Innovations. Education Sciences. 2024;14(6):616. doi: 10.3390/educsci14060616

[92] Howard SK, Tondeur J, Siddiq F, Scherer R. Ready, set, go! Profiling teachers’ readiness for online teaching in secondary education. Technol Pedagogy Education. 2020;30(1):141–58. doi: 10.1080/1475939x.2020.1839543

[93] Amoako I, Anane E. Digital teaching competence and resilience across tutors of different age and gender in Ghana. Discov Educ. 2025;4(1). doi: 10.1007/s44217-025-00484-9

