# Supplementary material for: Exploring the link between AI usage intention and digital competence among college PE teachers: A moderated mediation model based on SCT and UTAUT
Source: PLoS One. 2025 Nov 21;20(11):e0334699. doi: 10.1371/journal.pone.0334699 (PMC12637994; doi:10.1371/journal.pone.0334699)
Supplement: S1 Table — (DOCX) [file pone.0334699.s001.docx]

**S1 Table. Measurement items (in English and Chinese language) adopted.**

| **Factors** | **Code** | **Items** |
| --- | --- | --- |
| Behavioral  intention | BI_1 | I intend to use AI technology in my future physical education teaching |
|  |  | 我打算在未来体育教学中使用AI技术 |
|  | BI_2 | I predict I would use AI technology in my future physical education teaching |
|  |  | 我可能在未来体育教学中使用AI技术 |
|  | BI_3 | I plan to use AI technology in my future physical education teaching |
|  |  | 我准备在未来体育教学中使用AI技术 |
| Social  influence | SI_1 | A colleague or peer thinks I should use AI technology in teaching |
|  |  | 同事或同行认为我应该在教学中使用AI技术 |
|  | SI_2 | A student or friend thinks I should use AI technology in teaching |
|  |  | 学生或朋友认为我应该在教学中使用AI技术 |
|  | SI_3 | My supervisors have been supportive of my use of AI technology in teaching |
|  |  | 我的领导在我使用AI技术方面提供了帮助 |
|  | SI_4 | My university or college encourages and supports me in using AI technology in teaching |
|  |  | 学校或学院支持并鼓励我在教学中使用AI技术 |
| Self-efficacy | SE_1 | I am confident of using AI technologies in physical education even if there is no one around to guide me |
|  |  | 即使没有人指导，我也很有信心将人工智能技术应用于体育教学 |
|  | SE_2 | I am confident of using AI technologies in physical education even if I have never used them before |
|  |  | 尽管我以前从未使用过人工智能技术，但我对将其应用于体育教学充满信心 |
|  | SE_3 | I am confident of using AI technologies in physical education even if I have only the manuals for reference |
|  |  | 即使只有手册可供参考，我也对将人工智能技术应用于体育教学充满信心 |
| Teachers’ digital competence | TDC_1 | I know how to transfer AI-generated teaching materials across devices or cloud platforms for physical education |
|  |  | 我知道如何在设备或云端之间传输AI生成的体育教学资源 |
|  | TDC_2 | I know how to use advanced videoconferencing tools to support AI-assisted physical education teaching |
|  |  | 我知道如何使用视频会议工具支持AI辅助的体育教学 |
|  | TDC_3 | I know how to adapt AI-generated content for use in physical education teaching |
|  |  | 我知道如何调整AI生成的内容以应用于体育教学中 |
|  | TDC_4 | I know how to check the security of AI tools or platforms used in teaching |
|  |  | 我知道如何判断教学中使用的AI工具或平台是否安全 |
|  | TDC_5 | I can solve AI-related teaching problems by searching online |
|  |  | 我能通过网络查找方法解决AI教学中遇到的问题 |
|  | TDC_6 | I can effectively search, evaluate, and use AI-related information for physical education teaching |
|  |  | 我能有效搜索、评估并使用与AI教学相关的信息 |
|  | TDC_7 | I can collaborate with colleagues using digital tools to support AI-integrated physical education teaching |
|  |  | 我能使用数字工具与同事协作开展AI融合的体育教学 |

**S1 Table. Continued.**

| **Factors** | **Code** | **Items** |
| --- | --- | --- |
|  | TDC_8 | I can create digital content using AI tools for physical education teaching |
|  |  | 我能借助AI工具创作用于体育教学的数字内容 |
|  | TDC_9 | I understand how to ensure digital safety when using AI technologies in physical education teaching |
|  |  | 我了解在体育教学中使用AI技术时如何保障数字安全 |
|  | TDC_10 | I can solve practical problems and support others in using AI technologies in physical education teaching |
|  |  | 我能解决体育教学中使用AI技术遇到的实际问题，并帮助他人提升相关能力 |
